# Prize Essay—Medical Association of the State of Alabama

**Published:** 1875-06-15

**Authors:** Benj. H. Riggs

**Affiliations:** Sec. M. A. S. A., Selma, Ala.


PRIZE ESSAY—MEDICAL ASSOCIATION OF THE STATE OF ALABAMA.—At the Annual Session of this Association, held at Montgomery during the second week in April, 1875, Dr. S. D. Seelye, of Montgomery, after some remarks regarding the increasing frequency with which cases of Bright's Disease are brought under medical care in this section, the unsatisfactory though extensive literature of the subject, and the generally fatal prognosis which we are obliged to give, rendering it one of the opprobria of the profession ; and believing that there is still some hidden light that may be made to illumine the subject if attention and inquiry are turned especially to it, proposed to place in the hands of this Association a prize of one hundred dollars for the best treatise on this disease. The proposition having been accepted by this Association, it now invites from the thinkers and investigators of the profession at large, the generous competition which its author invokes. BENJ. H. RIGGS, M.D.,
Sec. M. A. S. A., Selma, Ala.

